# Exploring the Impact of Different Milling Parameters of Fe/SiO_2_ Composites on Their Structural and Magnetic Properties

**DOI:** 10.3390/ma17040862

**Published:** 2024-02-12

**Authors:** Tetiana Rudeichuk, Denisa Olekšáková, Robert Maciaszek, Waldemar Matysiak, Peter Kollár

**Affiliations:** 1 Institute of Manufacturing Management, Faculty of Manufacturing Technologies, Technical University of Košice, Bayerova 1, 08001 Prešov, Slovakia; denisa.oleksakova@tuke.sk; 2 Institute of Physics, Faculty of Science, Pavol Jozef Šafárik University in Košice, Park Angelinum 9, 04154 Košice, Slovakia; robert.maciaszek@student.upjs.sk (R.M.); peter.kollar@upjs.sk (P.K.); 3 Institute of Materials Technology, Faculty of Mechanical Engineering, Poznan University of Technology, Piotrowo 3, 60-965 Poznan, Poland; waldemar.matysiak@put.poznan.pl

**Keywords:** soft magnetic composites (SMCs), compacted powder, surface smoothing, mechanical ball milling

## Abstract

This research focuses on the production process of soft magnetic composites in the form of 3D bulk compacts made from insulated powder particles using different milling parameters, aiming to enhance their magnetic properties and to study an innovative method of the powder surface “smoothing” technique. A structure analysis using scanning electron microscopy (SEM), EDS, and optical microscopy is also included. We found out that the samples made by the innovative method have lower density values. This can be caused by a more consistent SiO_2_ insulation layer on highly pure iron powder particles. A correlation between the mechanical smoothing method and better insulation of powder particles can help to provide eco-friendlier solutions for the preparation of soft magnetic composites, such as less usage of reagents and more consistent coverage of powder particles with lower final insulation thickness. The magnetic properties of these compacts are evaluated by coercive field, permeability, and loss measurements. The particle-level smoothing technique in some cases can reduce the value of coercivity up to 20%. For some samples, the ball-to-powder ratio has a bigger impact on magnetic properties than surface treatment, which can be caused by an increased amount of insulation in the SMC compacts.

## 1. Introduction

Today’s society is becoming increasingly dependent on a variety of electronic devices, such as sensors, computers, electromotors, inductors, electromagnetic circuits, etc. These devices contain different groups of magnetic materials, which are expected to have the best possible properties with minimum investment [1,2,3]. That is why researchers all over the globe are trying to meet the need by creating new alloy compositions or different composites and even their preparation can have a significant influence on their final magnetic properties [4,5].

Magnetic materials play an important role in various industrial applications, such as materials engineering, automotive systems, power generation, and electronic and electromechanical equipment. Ferromagnetic materials can be divided even further by their other magnetic properties such as coercivity. Depending on their coercivity, ferromagnetics are divided into two main groups, namely, hard magnetic materials (HMMs) and soft magnetic materials (SMMs). The main difference between these two groups is their coercivity value, which affects the total amount of energy needed for one demagnetization process and the shape of the B–H hysteresis loop. In the case of HMMs, they have a wider hysteresis loop caused by a bigger value of coercivity (Hc), which makes them a great option for use as part of information storage devices in computer industries, magnetic filters, electric motors, and other applications where it is important for the material to have a high resistance to magnetization process.

Soft magnetic materials exhibit interesting characteristics like low coercivity, high permeability, and low energy losses. Modern and contemporary material research indicates a constant expansion of the achievable range of magnetic properties in soft magnetic materials. Compacted soft magnetic materials, derived from powder compaction and falling within the extensive category of such materials, find application in diverse electromagnetic scenarios. These include magnetic circuits for valves, electromotors, and cores in inductors for relays, as well as in devices like disk drives, computers, printers, and hearing aids [6,7,8].

Soft magnetic composites (SMCs) represent a fast-growing subgroup of soft magnetic materials requiring relatively low investments for their production. In most cases, these materials are made from ferromagnetic powders milled in different types of mills under different conditions, which can be easily changed by using a different ball-to-powder ratio (BPR) [9,10,11]. The milled powders can be coated with an organic, inorganic, or organic–inorganic coating to reduce eddy current loss. When ferromagnetic powders are prepared by milling, it can result in a lot of inner structural defects, which also have a high effect on the final magnetic properties of the SMC as well [9,12]. Consequently, to eliminate the influence of the milling process, powders can be annealed at high temperatures. As a result, an inorganic coating is a better option than an organic coating because organic insulation has a much lower decomposition temperature. Different oxides such as SiO_2_, Al_2_O_3_, and TiO_2_ usually can appear as inorganic insulation for iron-based SMCs [1,13,14,15]. Then, these insulated powders are compacted into different shapes depending on their future application, so their production is based mostly on conventional powder metallurgy and can produce relatively large amounts of materials in a small amount of time [16]. Accordingly, the used materials influence the final cost and the final properties of the compacted composite.

We can consider compacted samples made from a combination of different fractions of the same material, a combination of different soft magnetic materials, and insulated powder particles as SMCs [17,18]. The advantage of soft magnetic composites is that they can be compacted in various shapes, which can lead to a reduction in the amount of waste in bigger productions. The choice of materials depends on the desired magnetic properties and a lot of the SMMs are sold by companies with promised values of physical quantities such as coercivity (H_c_), permeability (µ), and Curie temperature [19,20].

From a theoretical point of view, the type of the SMCs made from compacted, randomly oriented, and insulated ferromagnetic powder particles could significantly reduce the amount of energy needed for the magnetization process and the magnetization reversal process. In that case, insulation is used for reducing the size of the domain walls, so less amount of energy is needed for changing the orientation of each domain [20].

Adding different materials into composites could have an influence on the final performance as well as the milling condition of sample preparation. For example, adding Cr can improve corrosion resistance and the mechanical strength of the material [21]; depending on the nickel percentage in Fe-Ni alloys. Final products can be used for high-frequency devices, or in cases requiring a high level of the initial permeability; Co in Fe-Co alloys enables a high saturation density to be expected, and consequently they can be used for the motors of high-speed generators [22,23]. Depending on the expected use of the SMCs, we can choose the best possible material from already existing products in the market, like powder iron developed for Somaloy or alloys such as permalloys (FeNi), supermalloys (NiFeMo), and Vitroperm (based on Fe with different percentages of Si, B, Nb, and Cu) [20,24,25].

These Fe-based materials exhibit favourable magnetic and mechanical properties, but they still offer the possibility to improve them. One of the ways is the innovative powder particle modification method that significantly influences magnetization processes in compacted materials prepared from treated surface powder particles (smoothing process) [6,26]. We investigated this smoothing process on supermalloys in [27]. We found that the compacted sample prepared from smoothed powder particles exhibits lower coercivity, higher initial and maximum relative permeability, and low frequency core losses in comparison with compacted samples prepared under the same conditions from non-smoothed particles [27].

This article presents how the preparation process affects the magnetic properties of soft magnetic composites (SMCs). We expect the smoothing process to have a prevailingly positive and only slightly negative influence on the preparation of the powders by the smoothing process. We assume that the positive effect of the smoothing process is that surface irregularities hindering the displacement of the domain walls will be partially removed. The negative influence can be the introduction of dislocations (structural defects) on the surfaces of thin layers by mechanical processing and it can negatively influence the domain wall displacement.

## 2. Materials and Methods

To study the structural and magnetic properties, we prepared six samples from highly pure iron granules (Alfa Aesar, Karlsruhe, Germany, 1–2 mm, and 99.98% purity) under different conditions of their preparation process.

Even if the main purpose of this article was to study the influence of the surface smoothing method, we were also able to compare the influence of the different values of the ball-to-powder ratio (BPR) on the final magnetic properties [26,27]. The reasons for choosing iron granules were their suitable magnetic properties and price [22,23,28].

### 2.1. Mechanical Milling (Preparation of the Powder)

Firstly, to prepare the powder with a needed fraction, the granules were milled in the Retsch PM100 planetary ball mill (Retsch, Haan, Germany) with different ball-to-powder ratios for 120 min with a 10 s break for stabilization of the material and mill temperature and with reverse rotation in each cycle for a duration of 70 s for optimal mixing and a consistent milling process. The milling speed was 500 rpm.

### 2.2. Sieving of Powdered Particles (Separation of the Suitable Size Fraction)

After the milling process, the powder was sieved and a fraction under 400 µm was chosen. In theory, the smaller particles could fill the gaps created between bigger particles during the compaction process and consequently, the compacts would have a larger density volume and less amount of air voids [29]. Then, the sieved powder particles made by using different BPR values were divided into two groups, one of which used a relatively new method of the surface “smoothing” process.

### 2.3. Smoothing Process (Mechanical Treatment of Surfaces of Powder Particles)

Mechanical treatment was made in the same planetary ball mill, but without grinding balls. On the grinding jar walls, an abrasive paper (Carborundum Electrite a.s, Benátky nad Jizerou, Czech Republic, with grain size 1000 p/mm^2^) was glued using Pattex Chemoprene Extrem (Henkel AG & Co. KGaA, Dusseldorf, Germany) and then the powder was put into that jar for mechanical treatment. This process lasted for 70 min with the same parameters as the milling process (500 rpm, a 10 s break each minute, and a reverse rotation).

### 2.4. Insulation (Preparation of the Precursor for Soft Magnetic Composites)

The next step in the SMC preparation was insulation to prevent “particle–particle” electric contact. Each powder sample was separately insulated by inorganic insulation using the Stöber method [26]. This “wet” method of insulation can create a consistent layer of insulation on the powder particle surface by a chemical reaction during mixing isopropyl alcohol (320 mL), distilled water (64 mL), tetraethyl orthosilicate (TEOS, 98%, 32 mL), and ammonia (8 mL) for coating 10 g of the iron powder. The insulation process was made in two sessions for a total time of 16 h, 8 h for each session. At the end of the insulation process, a high-purity iron powder would have a thin layer of SiO_2_ [23,30].

### 2.5. Compaction (Preparation of Compacted Soft Magnetic Composites)

The SMC powder (3.7 g) was poured into the matrix and then the final bulk samples S1–S6 were compacted into a ring-shaped composite with an inner diameter of 18 mm and outer diameter of 24 mm in a pressing apparatus with a pressure of 700 MPa for 3 min at a temperature of 400 °C in a vacuum. The entire heating process up to 400 °C took about an hour for each sample and then the cooling process took up to six hours.

**Figure 1:**
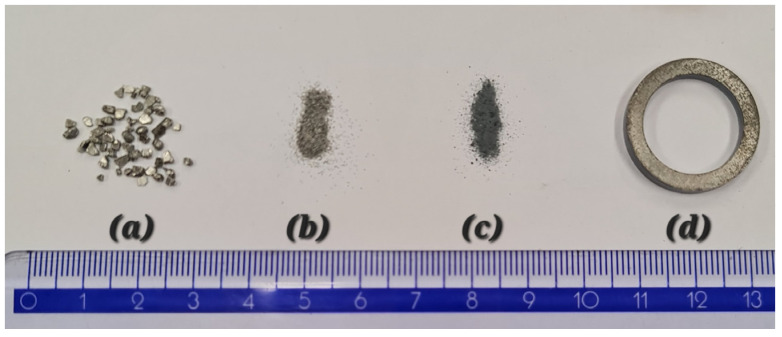
(**a**) Highly pure iron granules (1–2 mm) by Alfa Aesar, (**b**) milled iron granules with a fraction under 400 µm, (**c**) iron powder with SiO_2_ layer, (**d**) SMC bulk samples prepared by compaction.

The whole process of the SMC preparation is summarized in Figure 1, where we can see iron granules being milled and then insulated (the powder in this case seems to be a little bit lighter).

### 2.6. Conditions for Measurement Methods

Table 1 presents the technical characteristics as well as the conditions of the sample preparation process. The values of density of the samples represented in Table 1 were calculated from the weight and dimensions of the final bulks.

The coercivity values of the compacted SMC samples as well as the powder before compaction were measured using Foerster KOERZIMAT 1.097 HCJ, Reutlingen, Germany.

The dc and ac hysteresis loops in the frequency range from 100 Hz to 1000 Hz for the compacted samples were measured by the permeameter AMH-1K, Laboratorio Elettrofisico, Nerviano, Italy. The primary coil had 700 turns and the secondary coil had 200 turns for each measured sample. The maximum magnetic induction for the measurement was set at 0.1 T. The values of coercive fields and energy losses were evaluated from the measured hysteresis loops (with an accuracy of 3%).

## 3. Results

### 3.1. Morphology

**Figure 2:**
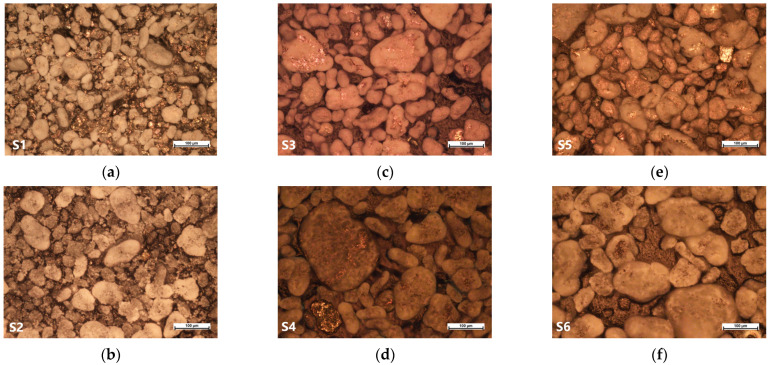
SMC images (with 20× magnification) of the highly pure iron powder particles with an SiO_2_ layer before the compaction, where (**a**) sample S1, (**b**) sample S2, (**c**) sample S3, (**d**) sample S4, (**e**) sample S5, (**f**) sample S6.

To analyse the structural properties of powder SMCs, we chose the optical microscope Nikon Eclipse MA200 (Nikon, Tokyo, Japan) (Figure 2). Images taken at 20× magnification show that insulation layer is a little bit more even on the samples with the mechanical treatment technology. Consequently, it decreases the chances of creating larger domain sizes that need less energy because of the localized intra-particle eddy current created around domain wall movement. After further investigation, these correlations can probably reduce the time for the insulation process or even reduce the number of reagents needed for the insulation process for particles with smoothed surfaces (S2, S4, and S6) compared to non-smoothed ones (S1, S3, and S5). In that case, SMCs made by surface smoothing process have a possibility to become an eco-friendlier method than the convection one.

Table 1 shows that the density of the material compacted from smoothed powder particles (S2, S4, and S6) is much lower than that of other samples made without surface treatment (S1, S3, and S5). This can be caused by a bigger amount of SiO_2_ on the surface because the density of SiO_2_ has a lower value than the density of pure iron. This can be used as one of the indirect types of evidence for the earlier statements that compacts made of “smoothed” powder particles have better layers of insulation.

The SMC compacted samples S1, S3, and S5, made from irregular particles, display a notably higher density compared to their “BPR-equivalent” (S2, S4, and S6) compacted from more smoothed powder particles with a theoretically more “spherical” shape, as shown in Table 1. This significant variation in density observed between the samples can also be described by the complexities during the compaction process. As a result, the irregular particles of the S1, S3, and S5 samples undergo breakage of the powder particles, which can create small fragments that can effectively fill the interparticle gaps. Consequently, it results in a higher overall density due to the effective positioning of the broken powder pieces. On the other hand, we can suppose that the breakage of the inner particle during compaction could possibly have a negative influence such as higher local pressures, which can affect the inner material structure. These local pressures increase the number of structural defects that influence domain wall displacement. The presence of these structural defects is correlated with the degradation of the soft magnetic properties of the compacts. On the other hand, the inner-particle gaps between smoothed particles in S2, S4, and S6 SMC compacts are probably larger, leading to a slightly lower density. This assumption suggests a more homogeneous distribution of the pressure on the particles during compaction. However, the larger interparticle gaps in these samples may create the potential for a more substantial inner demagnetizing field when the bulk is magnetized, introducing another layer of complexity in the magnetic behaviour of the material.

**Figure 3:**
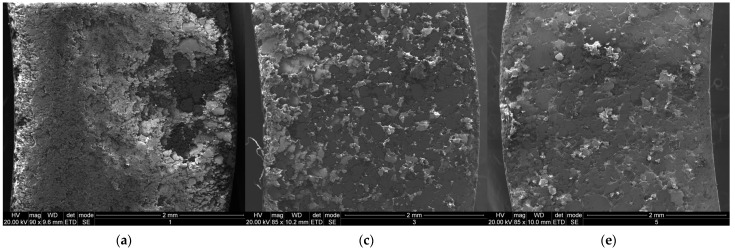

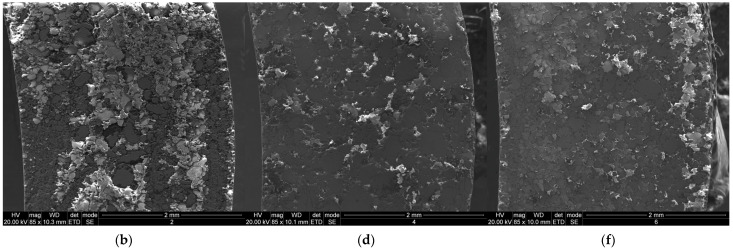
SEM morphology of the outer surface for the ring-shaped Fe/SiO_2_ compacts (**a**) sample S1, (**b**) sample S2, (**c**) sample S3, (**d**) sample S4, (**e**) sample S5, (**f**) sample S6.

Scanning electron microscopy (SEM) images (Figure 3) were made of the ring-shaped bulks (upper, inner, and outer side surfaces) before they were prepared for magnetic measurements. Pictures were taken of the top surface of the ring-shaped compacted bulks (position on the medium radius). These images show the flatter surface and slightly less amount of air voids in the structure of the ring-shaped compacts S2, S4 and S6 in comparison with S1, S3, and S5, the ferromagnetic powder particles of which did not have surface smoothing before. Even with a smaller amount of air voids, the density (Table 1) of S2, S4, and S6 has a lower value than their respective “BPR-equivalent” (S1, S3, and S5), which also can be described by a more consistent insulation layer on the powder particles before compaction. It can be noticed that the surface as well as its inner and outer sides for the SMC compacts (S2, S4, and S6) are flatter than those of “non-smoothed” samples (S1, S3, and S5).

**Figure 4:**
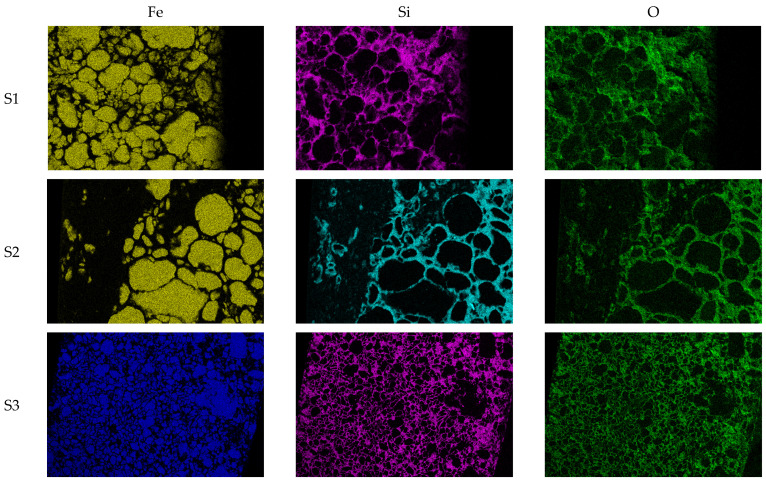

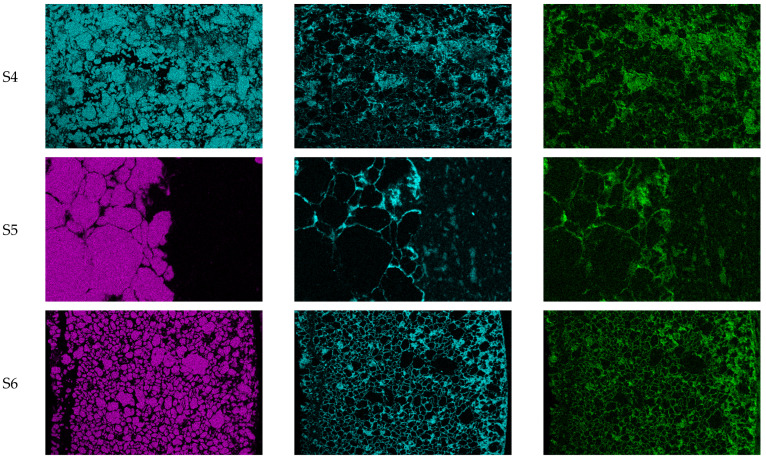
EDS spectrum of the surface for the outer surface for the ring-shaped Fe/SiO_2_ compacts (S1–S6).

For visualization purposes of the consistency of the insulation layers, we generated energy dispersive spectroscopy (EDS) images using SEM, which can be seen in Figure 4. It shows the EDS of ring-shaped soft magnetic compacts by highlighting the iron powders as the core of the SMCs and silicon and oxygen as components for the insulation layers. It can be noticed that the individual iron powders are better insulated if the compact is made from powders with surface treatment. It is clearly seen that silicon created a more consistent mesh around the powder particles, which prevented the iron–iron touch. Unfortunately, images that represent the oxygen location also show signs of iron oxidation.

The X-ray diffraction was also made for all six samples and XRD diagrams confirmed that the iron powder particles have a cubic crystal system for all the samples.

### 3.2. Magnetic Properties

We can see in Table 2 that the value of coercivity decreases with the increase in the BPR. The monotonous decrease in coercivity with the BPR for compacted samples is because the higher BPR leads to the smaller mean size of the powder particles that are less deformed at the next compaction, which leads to a lower probability for the creation of structural defects during mechanical milling for domain wall displacement. The great impact of the surface smoothing process could be seen at BPR 3:1 for S1 and S2, when between S3 and S4 with BPR 6:1 or S5 and S6 with BPR 9:1, the coercivity values were not that much different.

**Figure 5:**
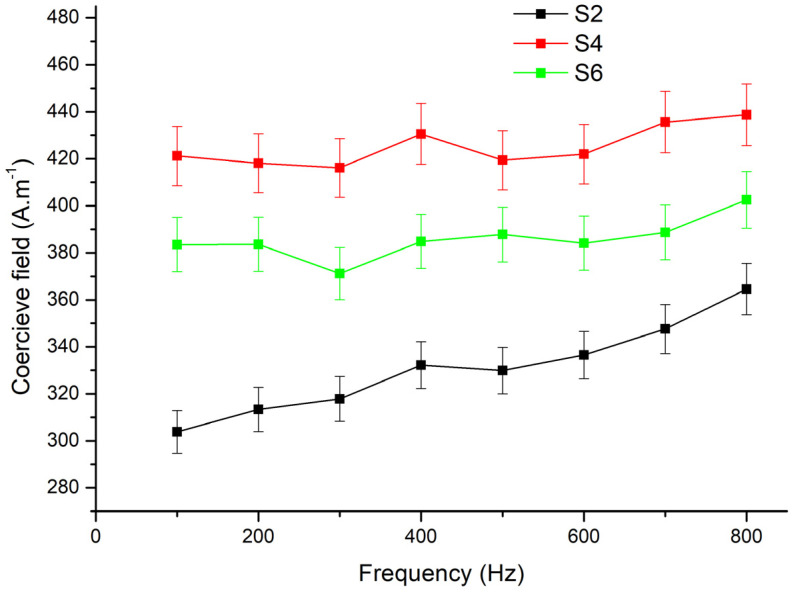
The coercive field of the Fe/SiO_2_ SMC compacts at different frequencies.

**Figure 6:**
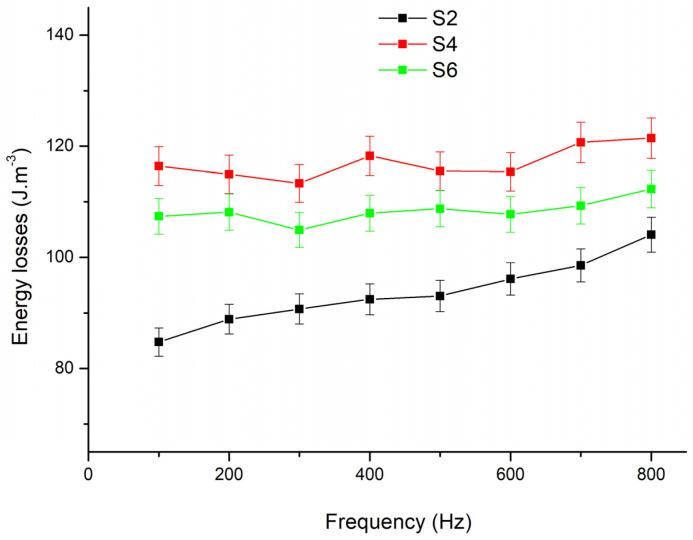
The energy losses of the Fe/SiO_2_ SMC compacts at different frequencies.

For further research of magnetic properties, we decided to investigate samples S2, S4, and S6 (prepared by the smoothing process) because of their lower coercivity values compared to samples S1, S3, and S5 (Table 2) and thus better magnetic properties for soft magnetic materials. We measured the coercivity field (Figure 5) and energy loss (Figure 6) values during increasing frequency levels from 100 Digittal FLUX by Laboratorio Elettrofisico Engineering s.r.l. Hz up to 1000 Hz. The measurements were made by the fluxmeter (Laboratorio Elettrofisico Engineering s.r.l., Nerviano, Milano, Italy) The samples S4 and S6 show relatively stable responses with a slight rise in the coercive field and losses in reaction to an increase in the frequency level. Sample S2 exhibits a slightly lower coercive field and losses vs. frequency compared to S4 and S6. As the frequency increases, these differences become less noticeable.

We measured the core energy loss dependence on the frequency for SMC compacts. The measurements were made using the same fluxmeter as in previous measurements. The precision of the energy losses was 3% for each sample measurement. Figure 6 shows the values of energy losses for S2, S4, and S6 samples in the frequency range from 100 Hz to 1000 Hz. From the data we gathered, it is clear that the S2 sample with a BPR of 3:1 exhibits the most favourable outcomes. These results could be caused by the smaller quantity of milling balls during the mechanical milling process. The reduced number of milling balls likely resulted in less internal structural damage compared to samples prepared with higher BPR values. It can also be noticed that with increasing frequency the differences in energy losses between the samples become less noticeable. Meanwhile, S4 and S6 samples demonstrated greater stability as the frequency increased, in contrast to the S2 sample, which experienced a significant increase. The results suggest that S4 and S6 are more resilient to changes in frequency compared to the less stable behaviour observed in the S2 sample.

The coercive field and energy losses have a similar trend with decreasing frequency. As we can see, the coercive field and energy losses increase with the frequency. Sample S2, prepared by compaction of the powder (with smoothed powder particles milled with a lower BPR), exhibits lower values of losses and coercive field than samples S4 and S6 (milled with a higher BPR). It is probably related to changes in the dynamics of the magnetization process in sample S2 vs. samples S4 and S6; in samples S4 and S6, the edges on the particle surfaces act as obstacles for domain wall displacement.

It is noticeable that, in general, the values of the coercive field and losses are the lowest for sample S2, higher for sample S6, and the highest for S4. This fact can be explained by two mechanisms: first one is the influence of the increase in the BPR parameter on the density of the resulting compact and the second one is the size of the particles [31]. The BPR at the level of 6:1 will bring the milled powder to a state where the compacted samples (S4 and S6) have an equal density (Table 1). Probably, the particles of sample S4 were exposed to greater mechanical stress, leading to a greater concentration of structural defects on which the domain walls were hindered at the magnetization reversal process, which increased the coercive field and hysteresis losses, which also increased the total losses, which were higher than for sample S6.

**Figure 7:**
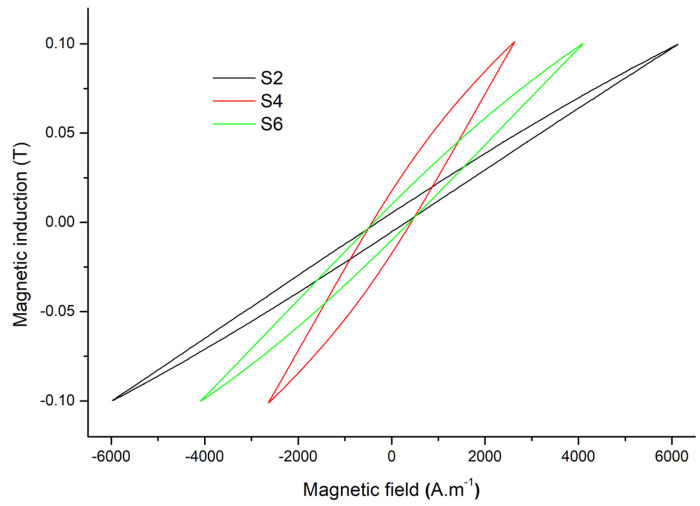
Hysteresis loops of samples S2, S4, and S6.

Figure 7 shows the dc hysteresis loops (for maximum induction of 0.1 T) for the samples made from surface-treated powder particles, which are S2, S4, and S6, to illustrate the particle-level smoothing treatment to support the coercive field and loss results described above.

## 4. Discussion and Conclusions

In soft magnetic composite materials based on iron with large magnetostriction, milling during powder preparation and mechanical treatment of the powder surface particles (smoothing process) can have a significant impact on the resulting magnetic properties of the composite [19,27,32,33].

In this work, we focused on investigating the influence of the conditions of mechanical milling of iron powder particles and its subsequent treatment by smoothing surface irregularities. Measurements of the coercivity of the milled powders showed that the coercivity value was found in each powder with a smoothed surface compared to the powder milled under the same conditions without surface treatment. For this reason, powders with non-smoothed surfaces were discarded from further investigation. The powders prepared by milling in a planetary mill with the parameters BPR 3:1, 6:1, and 9:1 for 120 min were covered with an electro-insulating layer of SiO_2_ by the Stöber method. This “wet” method of insulation can create a consistent layer of insulation on the powder particle surface by a chemical reaction during mixing isopropyl alcohol (320 mL), distilled water (64 mL), tetraethyl orthosilicate (TEOS, 98%, 32 mL), and ammonia (8 mL) for coating of 10 g of the iron powder. The powder obtained this way were pressed at a uniaxial pressure of 700 MPa for 3 min at a temperature of 400 °C in a vacuum.

From the frequency dependences of the coercive field and losses, it was found that:

- the lowest values of the coercive field and losses were for the compacted sample prepared from the powder milled at BPR 3:1, when the powder particles were the least damaged in comparison with other samples;
- milling reduces the powder particles size and introduces defects, acting as obstacles for domain wall displacements, thereby leading to an increase in the coercive field and total losses;
- smaller particle sizes provide a perspective for a higher density of the resulting composite within a certain density limit;
- the particle size and the density of structural defects are important parameters to determine the magnetic properties of the resulting compacts;
- the best magnetic properties from a series of six samples were shown by the sample prepared by compacting the milled powder, which was created in a planetary mill at BPR 3:1, when the coercive field at a frequency of 500 Hz and maximum induction of 0.1 T was 330 A.m^−1^ and a total loss of 93 J.m^−3^.

We have found out that the positive influence of the smoothing process applied on the particle surface and the optimized BPR on soft magnetic properties can be explained by a reduction in the amount of surface and volume obstacles for domain wall displacement.

## Figures and Tables

**Table 1:** Conditions of the sample preparation process with the technical characteristics of the SMC compacts.

	S1	S2	S3	S4	S5	S6
BPR	3:1	3:1	6:1	6:1	9:1	9:1
Mechanical treatment of powder particles (“smoothing process”)	No	Yes	No	Yes	No	Yes
Inner diameter of the compressed sample, [mm]	17.907	17.917	17.920	17.876	17.892	17.893
Outer diameter of the compressed sample, [mm]	24.080	24.083	24.064	24.032	24.050	24.057
Height of a compressed sample, [mm]	3.489	3.832	3.067	3.390	2.917	3.429
Mass of a compressed sample, [g]	3.694	3.704	3.704	3.694	3.794	3.744
Density of a compressed sample, [kg·m^−3^]	5200	4750	6020	5380	6410	5380

**Table 2:** Coercivity values of Fe/SiO_2_ SMC samples.

Name of the Sample	S1	S2	S3	S4	S5	S6
Coercivity of milled powder before compaction [A.m^−1^]	2970	2370	1980	2000	1730	1720
Coercivity of compacted SMCs [A.m^−1^]	1910	1460	1240	1230	1160	1150

## Data Availability

The data presented in this study are available from the corresponding authors upon reasonable request.
